# “Lights and Shades” of Fertility Preservation in Transgender Men Patients: A Clinical and Pathological Review

**DOI:** 10.3390/life13061312

**Published:** 2023-06-01

**Authors:** Antonio D’Amato, Eliano Cascardi, Andrea Etrusco, Antonio Simone Laganà, Luca Maria Schonauer, Gerardo Cazzato, Antonella Vimercati, Antonio Malvasi, Gianluca Raffaello Damiani, Edoardo Di Naro, Giuseppe Trojano, Ettore Cicinelli, Amerigo Vitagliano, Miriam Dellino

**Affiliations:** 1 Obstetrics and Gynaecology Unit, Department of Biomedical Sciences and Human Oncology, University of Bari “Aldo Moro”, Piazza Giulio Cesare 11, 70124 Bari, Italy; antoniodamato19@libero.it (A.D.); luca.schonauer@uniba.it (L.M.S.); antonella.vimercati@uniba.it (A.V.); antoniomalvasi@gmail.com (A.M.); damiani14@alice.it (G.R.D.); edoardodinaro@uniba.it (E.D.N.); giuseppe.trojano@policlinico.ba.it (G.T.); ettore.cicinelli@uniba.it (E.C.); 2 Department of Medical Sciences, University of Turin, 10124 Turin, Italy; eliano.cascardi@ircc.it; 3 Pathology Unit, FPO-IRCCS Candiolo Cancer Institute, 10060 Candiolo, Italy; 4 Unit of Gynecologic Oncology, ARNAS “Civico—Di Cristina—Benfratelli”, Department of Health Promotion, Mother and Child Care, Internal Medicine and Medical Specialties (PROMISE), University of Palermo, 90127 Palermo, Italy; etruscoandrea@gmail.com (A.E.); antoniosimone.lagana@unipa.it (A.S.L.); 5 Department of Emergency and Organ Transplantation, Pathology Section, University of Bari “Aldo Moro”, Piazza Giulio Cesare 11, 70124 Bari, Italy; gerardo.cazzato@uniba.it

**Keywords:** fertility preservation, transgender men, gender-affirming surgery, gender-affirming hormonal therapy

## Abstract

Fertility preservation (FP) is becoming a critical issue in transgender men who desire biological offspring in the future. The prevalence of transgender individuals in the United States is increasing, and as a result, the demand for gender-affirming surgeries (GAS) and associated FP techniques is rising. Despite the growing demand, there is currently no personalized approach to FP for transgender men, and the available techniques have limitations that require further investigation. In the present review we carefully examine the existing literature on this topic to highlight the shortcomings of current methods and areas where additional research is needed to advance the field. Hormonal therapy (HT), which is an integral part of gender transition in transgender men, can have a significant impact on fertility and may increase the risk of various diseases. Moreover, GAS usually leads to permanent sterility in these patients. Therefore, it is essential to provide patients with accurate information about the benefits and potential risks of different FP techniques, taking into account the patient’s reproductive goals. This review underscores the complex and multifaceted nature of FP in transgender men and emphasizes the need for further research to develop more effective and personalized approaches to FP for this population.

## 1. Introduction

Fertility preservation (FP) represents an increasingly crucial aspect of healthcare nowadays for assigned female at birth (AFAB) individuals who want to become men and at the same time who wish to have their own biological offspring in the future. According to a recent demographic report, over 1.6 million people identify themselves as transgender in the United States (0.6% of population), and they account for 1.4% of young people between 13 and 17 years old [1]. The number of gender-affirming surgeries (GAS) being performed globally is rising [2], and with them also the need for more comprehensive research on the outcomes of these types of surgery. Furthermore, with the recent advances in medicine and easier access to information in this regard, the rate of demand for FP techniques is increasing constantly over the last few years, noting that the desire of building a family is not uncommon in these subjects, especially after gender-affirming therapy (GAT) [3]. Nevertheless, gaining access to FP counseling may be harder for these patients than for cisgender individuals; the reasons are manifold, such as financial barriers and lack of available services or knowledgeable healthcare figures [4,5]. More importance should be given to this aspect, considering also that transgender individuals could undergo significantly lower rates of successful FP compared to cisgender individuals [6]. All of the most eminent statements provided in the past years by the principal scientific societies agree that fertility counseling should be provided for all transgender individuals, and that this should be offered and eventually carried out before the start of the transition period [7,8,9,10]. As evidence of this, the 2015 ASRM Ethics Committee opinion emphasizes the importance of providing access to ART for transgender individuals who desire it but notes that the use of hormones as part of gender-affirming care can have an impact on fertility, and recommends that healthcare providers discuss the potential effects of hormone therapy on fertility with transgender patients [8]. Hormone therapy (HT) and GAS can impact reproductive health and may affect the success of FP procedures [8]. On the other hand, there is not a clear consensus and/or a standardized approach to the GAT [10], leaving patients free to request personalized and more conservative strategies, which are not always safer. The aim of this review is to thoroughly analyze the current literature, in order to highlight areas where further research is needed to fully understand the benefits and risks of fertility preservation in this population, including the potential impact of hormone therapy and surgery on fertility and on overall health, and the psychological and emotional impact of FP on transgender men individuals.

## 2. Materials and Methods

A comprehensive literature search was conducted on the following databases: PubMed/MEDLINE, SCOPUS, The Cochrane Library, Science Direct, and Web of Science. All the relevant studies published until February 2023 were screened. The search terms used were “transgender”, “fertility preservation”, “transgender men”, “gender dysphoria”, “hormone therapy”, “androgen therapy”, “oocyte cryopreservation”, “embryo cryopreservation”, “in vitro maturation”, “in vitro fertilization”, “pregnancy outcome”, “reproductive techniques”, “ovarian cancer”, “endometrial cancer”, and “breast cancer”. The Boolean operators “AND” and “OR” were used to combine search terms as appropriate. Additional articles that met the inclusion criteria were identified by searching through the reference lists of the included articles. Articles that were not written in English or did not focus on transgender men were excluded. The search was conducted independently by two reviewers, and further supervised by a third, reliable member of the research group. Any discrepancies were resolved by discussion. The data were extracted and summarized using a narrative synthesis approach.

## 3. Fertility Preservation Options for Transgender Men

The current options available for fertility preservation in transgender men are as follows:

- Oocyte/Embryo cryopreservation;
- Ovarian tissue cryopreservation (OTC) and in vitro maturation (IVM).

### 3.1. Oocyte/Embryo Cryopreservation

Oocyte or embryo cryopreservation is an established technique that can be performed before or after HT [8,9,10]. However, there is no unanimous opinion on the actual effects of androgenic therapy on fertility outcomes. The literature in this regard is not yet sufficient and clear. Recently, Israeli et al. retrospectively compared embryos from 7 testosterone-treated transgender men with a control group of 34 cisgender women, 10 of whom underwent embryo cryopreservation, and 24 of whom underwent fertility treatment. They found no significant differences between the transgender men and the cisgender women who preserved fertility in terms of the number of cryopreserved embryos or the distribution of embryo age at cryopreservation, while they found a significantly higher mean number of oocytes retrieved in the transgender group compared to the cisgender women fertility treatment group [11]. However, the follow should be noted: the heterogeneity of the two groups, the small sample size, the study design, and the younger age of the transgender men patient group. There is no clear consensus on the timing of controlled ovarian stimulation (COS) after the suspension of HT as well [8]. Steinle et al. suggest that HT should be discontinued for at least three months before starting COS, in order to return to more physiological hormonal levels, and to minimize the risk of ovarian hyperstimulation syndrome (OHSS) [12]. Other studies, however, report that the resumption of menses after the cessation of HT could be expected to occur after at least 6 months of discontinuation [13]. Interestingly, a recent case report involving two transgender men without suspension of GAT showed a total number of 39 metaphase II (MII) oocytes retrieved in the two patients [14]. Further studies with larger sample sizes and better designs are needed in order to upgrade the treatment protocols for AFAB patients. After COS, the oocytes obtained can be cryopreserved for future use, or fertilized with donor sperm or by a cisgender sperm partner, depending on the male or female partner of the patient. Similarly, the embryos eventually collected can be cryopreserved, or freshly transferred into a gestational carrier, in the uterus of the individual if not removed surgically, or in the uterus of the female partner, depending on the sex of the partner. Ovarian or embryo cryopreservation are standardized treatments widely used in the field of Assisted Reproductive Technology (ART) and, thus, currently represent the treatments of choice for FP in transgender patients [9,10,15]. Despite this, both techniques could cause discomfort to the subjects, mainly due to the following side effects: the need to suspend HT in order to start COS; the process of COS itself can bring both physical changes and psychological distress; and frequent vaginal ultrasound monitoring may not be easy to bear for these patients [16]. All these factors can negatively impact the FP journey, increasing the chances of FP rejection and/or drop out.

### 3.2. Ovarian Tissue Cryopreservation and In Vitro Maturation

Ovarian tissue cryopreservation and in vitro maturation are two developing techniques for FP in transgender patients that are not as broadly spread among practitioners as oocyte or embryo cryopreservation. Nevertheless, these two methods of treatment could bring some benefits to transmen patients and, therefore, open new scenarios in the FP protocol.

#### 3.2.1. Ovarian Tissue Cryopreservation

OTC consists of surgical excision of part of the ovarian tissue or the whole organ and its cryopreservation for a future use. It is generally performed in laparoscopy under general anesthesia. The tissue picked up can be subsequently thawed, reimplanted into the patient’s body, and eventually stimulated to mature and produce viable oocytes at a later time. The possible advantages of this technique are represented by the possibility of not stopping the hormonal therapy for the patients; it could be performed at the same time as the GAS. Additionally, the eventual ovarian stimulation could be postponed until a later or further usage [15]. Furthermore, it constitutes the only viable option for FP in patients of prepuberal age. As a transplantation site, the orthotopic should be referred, rather than the heterotopic, for the higher success rate of the procedure [17]. Regarding the cryopreservation method, slow freezing is considered the standard method for human ovarian tissue, [10] but in recent times a number of studies have demonstrated positive outcomes through the utilization of the vitrification method [18]. More recently, Borrás et al. compared the two freezing methods in 18 AFAB individuals whom underwent OTC, demonstrating a similar accuracy in both procedures [19]. For subsequent handling and culturing, a diameter of the fragments of ovarian cortex of 4 mm appears to be the most suitable [20]. There is a lack of data in the literature about the technique and/or pregnancy outcomes in transgender patients, while it has been used with success in several cases of FP for cancer patients and Premature Ovarian Insufficiency (POI) patients. In particular, OTC represents the treatment of choice in prepubertal cancer survivors [21], and so far more than 130 babies are born from cisgender women across the globe thanks to this technology [22].

#### 3.2.2. In Vitro Maturation

IVM of the oocytes from the ovarian tissue excised for cryopreservation could represent an option in order to avoid the negative features of the following tissue retransplantation after OTC, such as the need for a second intervention carried out under general anesthesia, the interruption of HT, and the multiple vaginal ultrasound checkups required for COS [23]. In addition, most patients decide to undergo OTC in the course of GAS, making reimplantation not feasible and, therefore, an alternative strategy necessary. Oocytes at different stages of maturation retrieved from the surgically excised tissue are cultured in vitro for ripening, and then thawed for fertilization through intracytoplasmic sperm injection (ICSI). In 2017, Lierman et al. collected data from a total number of 259 MII oocytes retrieved after IVM, showing a normal spindle structure analysis and chromosomal alignment after vitrification [24]. However, a more recent publication from the same group involving oocytes collected from 83 transmen patients under testosterone treatment showed high aberrant cleavage patterns and early embryo arrest after fertilization, suggesting a low development capacity of in vitro matured oocytes in this group of patients [25]. At the state of ART, IVM remains an experimental procedure and further studies are needed to upgrade the technique.

#### 3.2.3. Fertility Preservation Outcomes in Transgender Men Patients

Oocyte cryopreservation has shown promising results among transgender men patients, as shown by mounting evidence. Leung et al. carried out a matched retrospective cohort study involving 26 AFAB patients under HT whom underwent FP between 2010 and 2018, compared with 130 cisgender controls [16]. The mean number of oocytes retrieved after COS was 19.9 ± 8.7, higher than the one in the control group (15.9 ± 9.6). Interestingly, the total dose of gonadotropins used in the transgender group was higher than the one in the cisgender group [16]. Amir et al. compared FP outcomes in nine adolescent transgender men patients before starting GAHT with thirty-nine cisgender patients whom underwent FP prior to cancer treatment [26]. The number of oocytes retrieved, of MII oocytes, and maturity rates did not show any significant differences between the two groups [26]. The same result was found by Yan et al. in a systematic review [27]. The amount of available evidence is still limited but may suggest that oocyte cryopreservation is an effective technique for these individuals which can lead to results similar to those of cisgender patients [26,27]. This observation is supported by the absence of statistically significant differences in the number of MII oocytes retrieved in the research cited, since the collection of high-quality oocytes is a crucial step in FP. Furthermore, evidence from the abovementioned studies showed that HT may not adversely affect the results of the technique, although more publications are needed.

The literature about FP outcomes after OTC/IVM is not as full of evidence. Lierman et al. performed a number of studies about IVM with different results, which have been discussed previously [24,25]. A very recent study by Christodoulaki et al. analyzed oocytes retrieved from the ovarian tissue of ovaries removed from 19 transgender patients whom underwent a bilateral oophorectomy from 2020 to 2022 [28]. The oocytes collected from transgender patients were subsequently in vitro matured, and compared with control in vitro and in vivo matured oocytes from cisgender patients whom underwent ART treatment for infertility. After IVM and ICSI, the rate of fertilized oocytes was higher in the control group than in transgender one. Following embryo development, the rate of day-5 blastocysts was significantly higher in the control group. Applying the experimental technique of Spindle Transfer (ST), the rate of day-5 blastocysts increased to similar levels of the cisgender group [28]. According to the authors, different cytoplasmic factors such as poor calcium release could be the explanation for poor embryo development of the oocytes retrieved from ovarian tissue in these patients; ST represents a promising technology in overcoming the issue [28].

## 4. Psychological and Emotional Impact of FP on Transgender Men Patients

Several surveys found that to face a fertility preservation journey and eventually a pregnancy could elicit gender incongruences and worsen gender dysphoria in transgender men [13,29]. This should be taken into consideration when offering counseling to these patients. Transgender patients already experience intense distress due to the sensation of being trapped in the “wrong body”, and unpleasant attitudes should be minimized in order to reduce these feelings [8], even though it is recognized that the emotional distress related to gender nonconformity does not constitute, in itself, a mental disorder [30]. As previously mentioned above, the FP process may be emotionally and psychologically challenging for an AFAB individual. COS often leads to the development of feminine secondary sex characteristics, such as breast tissue development, and, thus, to the worsening of gender transition distress. For these reasons, efforts should be made in all the stages of counseling to relieve the feeling of anxiety and worry [8]. This includes creating an appropriate and welcoming office environment, setting an inclusive and gender neutral doctor–patient relationship, and establishing a nondiscriminatory policy, including the use of proper pronouns and the supply of gender-free bathrooms and documentation [15,31].

## 5. Histopathological Changes in Genital Organs and in Breast after HT

Gender-affirming hormonal therapy (GAHT) is a standard treatment for transgender men to achieve the cessation of menstruation and male secondary sex characteristic development. Androgen hormones, typically testosterone, are used to suppress endogenous estrogen production, induce amenorrhea, and promote the development of masculine traits. While GAHT has been shown to be effective in achieving the desired physical changes, there is limited research on the potential long-term effects of GAHT on the reproductive organs, specifically the ovaries and uterus, and on mammary tissue.

The results of the studies evaluating the ovaries, breast tissue, and uteri of transgender men patients by histological or image diagnostic assessment are summarized in Table 1, Table 2 and Table 3.

## 6. Effects of Gender-Affirming Hormonal Therapy (GAHT) on the Ovaries

Several studies have investigated the effects of GAHT on the ovaries of transgender men patients from a morphological and histopathological point of view, leading to conflicting results [53,54]. Increases in collagenization of the tunica albuginea, stromal hyperplasia, and luteinization of stromal cells are among the most frequent findings in terms of differences from physiological cisgender patients’ ovaries [36,37]. Instead, the role of GAHT in the development of the polycystic ovary (PCO) phenotype and polycystic ovarian syndrome (PCOS) is still unclear. Many studies over the years showed histological signs of PCO in the ovaries of AFAB patients after surgical excision [36,37,43]. Chadha et al. in 1994 studied androgen receptor (AR) expression in the ovaries of transgender patients, PCOS patients, and the control, finding an enhanced expression of AR in the first two groups and supporting the hypothesis of PCOS as an androgen-mediated syndrome [41]. On the other hand, more recent publications show different results in this regard. Ikeda et al. retrospectively compared ovaries from transgender men patients under HT and cisgender patients with adnexal pathology, whom did not receive androgen treatment, with both groups undergoing salpingo-oophorectomy. The results showed a significantly higher rate of ovarian cortex thickening, collagen fibers hyperplasia, ovarian stromal hyperplasia, and stromal luteinization in the first group but with no statistically significant differences in the number of primordial, primary, preantral, and early antral follicles [44]. Furthermore, Caanen et al. assessed ovarian morphology by transvaginal 3D ultrasonography in 56 transgender men patients and in an 80 cisgender women control group, finding no significantly different rates of PCOM in the first group compared to the second [55]. It should be noted that both the previously mentioned studies found higher rates of atretic follicles in the transmen group [38,39]. Borrás et al. performed a preoperative transvaginal ultrasound and serum hormones evaluation and postsurgical histological analysis in 70 AFAB undergoing GAS after HT. Antral follicles were detected in most patients, without identifying the dominant follicle or corpus luteum; the histological analysis found the majority of follicles in the primordial stage and a small part the atretic stage, and luteinization of the stromal cells in more than half of the samples [56]. Additionally, anti-Müllerian Hormone (AMH) and Antral Follicle Count (AFC) level variations before and after GAHT are not clear. Evidence from a prospective Israeli study highlights that androgen treatment may lead to a slight decrease in serum AMH levels but within the normality range, without affecting AFC over time [57]. Other studies instead proved an overall decrease in AMH levels overtime [46], and no differences [58].

## 7. Impact of GAHT on the Uterus

Scientific papers analyzing the histopathological changes in the uterus before/after HT have also shown conflicting data. Miller et al. examined uteri from 32 AFAB patients whom underwent surgical sex reassignment after GAHT, showing marked cervical atrophy and endometrial atrophy in most samples [38]. Chadha et al. found preponderant endometrial inactivity in their sample [41]. In 2010, Grynberg et al. performed a pathological analysis of the genital organs of 112 male transgender patients undergoing a hysterectomy with bilateral salpingo-oophorectomy. All the patients received at least 6 months of HT before surgery. They found endometrial atrophy in 45% of the samples, and in the remaining part signs of endometrial activity [36]. In another series of 27 transgender men patients under GAHT compared with cisgender women of fertile and postmenopausal age an inactive, atrophic endometrium was found, similar to the menopausal one [49]. In a prospective study published by our group, a low-grade proliferative activity was instead found in all samples [45]. Endometrial inactivity was the most frequent piece of evidence in the sample studied by Khalifa et al. It should be noted that a smaller number of patients showed signs of proliferative (5/27) and secretive endometrium (2/27) despite HT [48]. In one of the studies with the largest sample by Grimstad et al. an active endometrium was found in the majority of the patients (65/94; 69.1%), and complex hyperplasia without atypia in one patient [50]. More recently, Hawkins et al. analyzed a sample of 81 transgender patients undergoing GAS after androgen treatment, finding either proliferative (40%) or atrophic (50%) endometrium [51]. Asseler et al. compared the endometrial thickness of 51 transgender men undergoing at least one year of HT before GAS with 77 controls. The median endometrial thickness in the first group was 3.9 mm, significantly lower than that of the control group (4.9 mm), suggesting that exogenous testosterone may not stimulate endometrial growth [52].

## 8. Impact of GAHT on Mammary Tissue

Testosterone therapy has been shown to induce significant changes in the breast tissue of transgender men individuals. The first histologic analysis was performed by Sapino et al. in 1990 on the udder of two transgender men patients subjected to long-term androgen therapy. Stromal sclerosis and atrophy of breast epithelium were the most relevant findings, together with small evidences of apocrine metaplasia, supposedly induced by HT [32]. Burgess et al. examined breast sections from 29 postmastectomy AFAB whom took HT for a prolonged period before the surgery, showing a significantly higher prevalence of microcalcification than the control, and no differences in the distribution of normal acini and ducts, cysts, fibrosis, and apocrine metaplasia, as well as progesterone and estrogen receptors [33]. Slagter et al. found an involution of breast tissue similar to the menopausal one in 23 patients, showing a significant decrease in the glandular tissue of lobuloalveolar structures together with and increase in fat deposition and fibrous connective tissue [34]. A very recent work by Ramos et al. focused on the changes in the diagnostic pattern. A mammography and breast ultrasound were performed in 33 transmen patients under GAHT for at least three years, highlighting an increase in the density of breast tissue compared to cisgender women [35]. Regarding a possible effect of GAHT on an increased risk of breast cancer, a systematic review of the literature analyzed ten retrospective cohort studies, finding a risk comparable to the cisgender women population. However, in the same study, nine cases of accidental breast cancer diagnosis encountered during postoperative histologic evaluation were reported [59]. Similarly, in an integration of three different systematic reviews, the poor-quality evidence included did not show an effect of HT on breast malignancy outgrowth [60]. Nevertheless, the same studies concluded that high-quality papers regarding the impact of GAHT in breast cancer development are lacking [59,60].

## 9. Discussion and Possible Future Approaches

Fertility preservation in transgender men patients is a complex and multifactorial issue that requires an individualized and multidisciplinary approach. It is important to take into account that despite several available and feasible options for FP, such as oocyte or embryo cryopreservation, these methods are often not accessible for AFAB patients [4,5]. The first consideration to be made is the timing of FP. The decision to pursue FP should preferably be made before starting HT or undergoing GAS, as these interventions can impact fertility potential [7,8,10]. However, some transgender men may delay FP due to financial or personal reasons, which highlights the need for improved access and education on the topic. However, another frequent reason not to pursue the FP path is the lack of adequate information provided by physicians, an aspect upon which efforts can and must be made [10]. Another consideration is the appropriate counseling before GAS. As stated above, the rate of GAS has been constantly increasing in the last years, due to different reasons. A growing number of transgender patients are receiving insurance coverage for GAS in the last years [61]. Female genital organs are often viewed as a symbol of femininity, which can cause significant psychological discomfort for some transmen [62]. Therefore, a hysterectomy with or without a bilateral salpingo-oophorectomy (BSO) can represent a solution to alleviate such discomfort and distress in some individuals with gender dysphoria. Ultimately, the recent progress of endoscopic surgery allows greater ease in the surgical approach; moreover, the uterus after prolonged HT is often small and involute, making it easier and faster to carry out the intervention by laparoscopy, thus performing a Total Laparoscopic Hysterectomy (TLH), with or without BSO [62]. The minimally invasive approach is characterized by less surgical bleeding and postoperative pain, better cosmetic result, and a shorter hospital stay. All this results in a higher satisfaction rate in transmen patients undergoing GAS. Despite this, patients should be carefully informed about the permanent infertility caused by the procedure before undergoing surgery [62].

On the other hand, it is not uncommon for patients to request different strategies, for instance asking to keep the uterus, or the ovaries, or in some cases the entire female genital system while taking GAHT. It is very important to provide the right information regarding the possible risks and benefits of retaining these organs. Saving the ovaries could enhance the endogenous testosterone production and prevent the cardiovascular and metabolic complications classically linked to hormonal deficiency. Saving the uterus, on the other side, can make it possible to carry a pregnancy in these patients.

**Figure 1:**
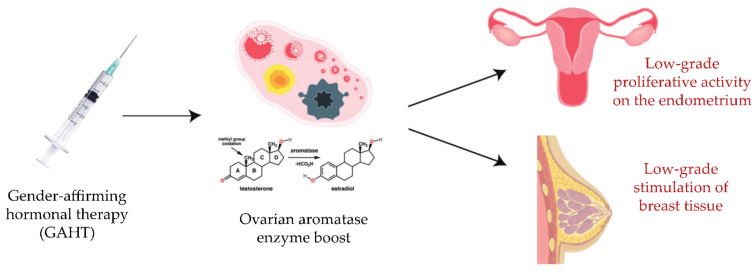
Effects of GAHT on endometrial and breast tissue: a hypothesis regarding the implication of aromatase in the pathogenesis of hormone-related malignancies in transgender men patients.

The current literature lacks clear evidence regarding the impact of GAHT, taken for decades in most cases, on endometrial, ovarian, or breast cancer development. A datum that cannot be neglected is the presence of the ovarian aromatase enzyme, through which a part of the androgen hormone can be converted into estrogen. In this regard, with the present review we would like to introduce a hypothesis regarding the implication of aromatase in the pathogenesis of hormone-related malignancies among these individuals. The long-term intake of androgen hormones, together with the persistence of aromatase activity, might involve basal ovarian and proliferative endometrial activity on one hand [45], and a slight but continual stimulation of breast tissue on the other, which could ultimately result in the development of a tumor (Figure 1).

In light of this and other mechanisms, it should be explained to patients that there is no literature in accordance in this regard, and that risks could occur; similarly, this information should be included in the informed consent signed by the patient during fertility counseling. At the same time, while evaluating the feasibility of a conservative approach it is equally important to investigate family history for genital tumors or genetic factors such as BRCA-1/2 mutations [63,64] or other gynecological alterations [65,66,67,68,69,70]. It is important, however, to note the speculative nature of our hypothesis, which requires high-quality evidence in order to validate or disprove it. We invite the investigators to carry out studies in this respect, to better define the role of residual enzymatic activity on the target tissues. If FP is an important consideration for many transgender men patients, it is also important to note that not all patients will choose to pursue FP. Some patients may not desire biological children, while others may prioritize other aspects of their transition. Hence, it is important for healthcare providers to provide information and support to patients while respecting their autonomy and decision-making capacities. Our review highlights the complexity and multifactorial nature of FP in transgender men patients. The decision to undergo FP should be tailored to the history and will of the patient, and made in consultation with a multidisciplinary team, including an endocrinologist, a fertility specialist, a mental health provider, and a gynecological surgeon. This approach ensures that the patient receives comprehensive care that takes into consideration their physical and mental health, as well as their reproductive goals. The multidisciplinary team can also provide valuable information and support to help patients make informed decisions regarding fertility preservation options.

## 10. Conclusions

In conclusion, the present review sheds light on the various aspects of fertility preservation in transgender men patients while also highlighting uncertainties. Firstly, the timing for the discontinuation of GAHT in order to optimize FP remains unclear. Further research is needed to determine the most appropriate approach in this regard. Secondly, it is evident that a multidisciplinary approach is essential for comprehensive fertility preservation in transgender men patients. Collaboration between endocrinologists, reproductive specialists, and mental health professionals is crucial to address the complex needs and concerns of these individuals. Lastly, the safety of preserving the uterus and ovaries in transgender men patients is still uncertain due to the potential effects of long-term testosterone treatment. Long-term studies are necessary to fully understand the risks and benefits associated with this approach. Overall, this review emphasizes the importance of ongoing research and collaboration in order to provide optimal fertility preservation options and care for transgender men patients.

## Figures and Tables

**Table 1:** Summary of findings from studies evaluating the breast of transgender men patients by histological analysis or transvaginal ultrasonography.

Study ID	Population	Control	Assessment	Average Duration of HT Intake	Findings
Sapino et al. [32]	2 transgender men	No control	Histologic	Mean: 30 months	Epithelial atrophy; in patient 2, apocrine metaplasia; and hyalinosis of periductal and interlobular stroma.
Burgess et al. [33]	29 transgender men	10 cisgender men patients with gynecomastia and 10 cisgender healthy women	Histologic	At least 18 months	Normal acini (28/29), normal ducts (29/29), fibrosis (29/29), cysts (13/29), apocrine metaplasia (15/29), epithelial hyperplasia (9/29), and microcalcification (8/29).
Slagter et al. [34]	23 transgender men	10 cisgender women	Histologic	18–24 months	Marked reduction in glandular tissue, involution of the lobuloalveolar structures, prominence of fibrous connective tissue, and reduction in fat tissue.
Ramos et al. [35]	34 transgender men	Expected results in cisgender women according to American College of Radiology—Breast Imaging Reporting and Data System (ACR BI-RADS^®^).	Mammography and breast ultrasound	Not available	Higher frequency of dense breast in transmen (66–6%) compared to expected results for cisgender women.

**Table 2:** Summary of findings from studies evaluating the ovary of transgender men patients by histological analysis or transvaginal ultrasonography.

Study ID	Population	Control	Assessment	Average Duration of HT Intake	Findings
Amirikia et al. [36]	10 transgender men	3 PCO and 3 cisgender women	Histologic	14 to 84 months (mean: 35 months)	Thickening of tunica albuginea and basal membrane (PCOS-like).
Futterweit et al. [37]	19 transgender men	12 age-matched cisgender women	Histologic	12 to 120 months (mean: 37 months)	PCOM (13/19), multiple cystic follicles (17/19), diffuse ovarian stromal hyperplasia (16/19), collagenization of the outer cortex (13/19), and luteinization of stromal cells (5/19).
Miller et al. [38]	32 transgender men	36 cisgender women	Histologic	12 to 96 months	Normal number of primordial follicles, follicular cysts, and corpora albicantia in all the patients; graafian follicles in a significant number of patients; current corpus luteum (1/32); and recent corpora lutea (3/32).
Spinder et al. [39]	26 Transgender men undergoing GAS	9 age-matched cisgender women	Histologic	9 to 36 months (mean: 18 months)	Normal primordial and developing follicles in all patients (26/26). Multicystic ovaries (18/26), collagenization of the tunica albuginea (25/26), diffuse stromal hyperplasia (21/26), luteinization of theca interna (18/26), luteinized stromal cells (7/26), presence of corpora lutea (4/26), and corpora albicantia (26/26). PCOM (18/26).
Pache et al. [40]	17 transgender men	13 cisgender women	Histologic	11 to 72 months (mean: 21)	Increase in macroscopic volume of the ovaries (11/29), thickened and collagenized ovarian cortex (28/29), primordial follicles (28/29), higher number of healthy antrals and atretic vs. control (27 ± 13 vs. 11 ± 5), healthy antral follicles (18/29), atretic follicles (29/29), theca interna hyperplasia (29/29), diffuse stromal hyperplasia (29/29), and luteinized stromal cells were organized in clusters (12/29).
Chadha et al. [41]	11 transgender men	10 cisgender women	Histologic	11 to 72 months (mean: 21)	Increased number of cystic follicles and atretic follicles, collagenized and thicker ovarian cortex, theca interna hyperplasia and luteinization of follicles, stromal hyperplasia accompanied by clusters of luteinized cells, and higher AR receptor expression in 11/11.
Mueller et al. [42]	45 transgender men	No control	Transvaginal ultrasonography	24 months	No significant changes.
Grynberg et al. [43]	112 transgender men	No control	Histologic	24 to 108 months (mean: 44)	Macroscopical enlargement (90/224 ovaries), stromal hyperplasia (112/112), PCOM (89/112), and strong correlation with mean ovarian volume and number of antral follicles.
Ikeda et al. [44]	11 transgender men	10 cisgender women with gynecologic malignancies who did not receive HT.	Histologic	17 to 164 months (mean: 70)	In transmen group, thicker ovarian cortex, more hyperplastic collagen, ovarian stromal hyperplasia and stromal luteinization, but similar number of primordial follicles and early stage (primary, preantral, and early antral) follicles in the two groups. Higher rate of atretic follicles in transmen group.
Loverro et al. [45]	12 transgender men	No control	Histologic	Mean: 32 months	Multifollicular ovaries (10/12) and corpora lutea presence (2/12).
Caanen et al. [46]	56 transgender men	80 cisgender women	3D transvaginal ultrasonography	>12 months	No significant differences in PCOM between transmen group (17/53—32.1%) and control group (23/75—30.7%).
De Roo et al. [47]	40 transgender men	No control	Histologic, in vitro maturation, and immunohistochemical	Mean: 14 months	1313 COC retrieved from the medulla of 35 patients; primordial follicles 68.52%, 20.26% intermediate, and 10.74%primary follicles. After 48 h IVM, 34.30% metaphase II oocytes obtained, with 87.10% having a normal spindle structure.
Khalifa et al. [48]	27 transgender men; 5 prior undergoing bilateral oophorectomy	No control	Histologic	24 patients received androgen from 19 to 288 months.	Bilateral cystic follicles (23/23) and higher follicular density.
Borràs et al. [19]	70 transgender men undergoing GAS after HT	No control	Preoperative transvaginal ultrasonography and AMH assessment, after surgery histologic evaluation.	Not available	Antral follicles (43/47), presence of dominant follicle or corpus luteum (0/47). Thickening of tunica albuginea and luteinization of stromal cells (68.6%). Negative correlation between testosterone levels and total antral follicles.

**Table 3:** Summary of findings from studies evaluating the uterus of transgender men patients by histological analysis or transvaginal ultrasonography.

Study ID	Population	Control	Assessment	Average Duration of HT Intake	Findings
Futterweit et al. [37]	19 transgender men	12 age-matched cisgender women	Histologic	12 to 120 months (mean: 37 months)	Proliferative endometrium (12/19), inactive (7/19), and leiomyomata (4/19).
Miller et al. [38]	32 transgender men	36 cisgender women	Histologic	12 to 96 months	Inactive endometrium (26/32), atrophic (6/32), and leiomyomata (5/32). Cervical mucosa atrophy (24/32) and focal interstitial eosinophilic infiltration (9/32)
Chadha et al. [41]	11 transgender men	10 cisgender women	Histologic and immunohistochemical	11 to 72 months (mean: 21)	Inactive endometrium (67% of samples), atrophic (33% of samples), and proliferative/active endometrium (0% of samples). AR expression more pronounced in myometrial and endometrial stroma in transmen group vs. control.
Mueller et al. [42]	45 transgender men	No control	Transvaginal ultrasonography	24 months	Lower endometrial thickness.
Perrone et al. [49]	27 transgender men	43 cisgender women, 13 fertile age, and 30 menopause	Histologic	12 to 72 months (mean: 34 months)	Inactive endometrium (27/27) and nonfunctional polyps (5/27). Ki67 expression in transmen group similar to the menopausal group; both lower than fertile women group.
Grynberg et al. [43]	112 transgender men	No control	Histologic	24 to 108 months (mean: 44)	Proliferative endometrium (54/112), atrophic endometrium (50/112), and leiomyomata (19/112).
Loverro et al. [45]	12 transgender men	No control	Histologic	Mean: 32 months	Proliferative endometrium (10/12), secretory endometrium (2/12), myometrial fibrosis (5/12), myometrial hypertrophy (2/12), and normal myometrium (5/12).
Khalifa et al. [48]	27 transgender men; 5 prior undergoing bilateral oophorectomy	No control	Histologic	24 patients received androgen from 19 to 288 months	Proliferative endometrium (5/27), secretory endometrium (2/27), inactive endometrial glands (20/27), and focal decidua-like endometrial stromal change (16/27); cervical metaplasia (ectocervical or in zone of transformation—17/27).
Grimstad et al. [50]	94 transgender men	No control	Histologic	0 to 12 months (22/94), >12 to 24 months (30/94), >24 to 48 months (19/94), and >48 months (23/94) Mean: 36.7 ± 36.6 months	Proliferative endometrium (61/94), atrophic endometrium (23/94), secretory endometrium (4/94), endometrial polyps/myomas (9/94), adenomyosis (7/94), complex hyperplasia without atypia (1/94), and other benign disease (4/94).
Hawkins et al. [51]	81 transgender men	No control	Histologic	Mean: 48 months (duration of preoperative HT noted only on 70 patients)	Proliferative endometrium (33/81), atrophic endometrium (40/81), endometrial polyps (9/81), and no cases of endometrial hyperplasia or malignancy.
Asseler et al. [52]	51 transgender men	77 cisgender women	Transvaginal ultrasonography	Mean: 30.2 months	Significantly lower endometrial thickness in transmen group compared with cisgender women: median 3.9 mm (interquartile range [IQR] 2.8–5.1) and 4.9 mm (IQR 4.0–6.3), respectively (*p* < 0.001), after correcting for confounding factor.

## Data Availability

Not applicable.
